# Novel synthesis of new triazine sulfonamides with antitumor, anti-microbial and anti-SARS-CoV-2 activities

**DOI:** 10.1186/s13065-024-01164-9

**Published:** 2024-03-26

**Authors:** Reham A. Mohamed-Ezzat, Galal H. Elgemeie

**Affiliations:** 1 Chemistry of Natural & Microbial Products Department, Pharmaceutical and Drug Industries Research Institute, National Research Center, Cairo, Egypt; 2 Department of Chemistry, Faculty of Science, Helwan University, Helwan, Cairo, Egypt

**Keywords:** Anti-Microbial, Anti-proliferative, SARS-CoV-2, Triazines, Sulfonamides, Synthesis

## Abstract

Novel approach for synthesizing triazine sulfonamide derivatives is accomplished via reacting the sulfaguanidine derivatives with *N*-cyanodithioiminocarbonate. Further reaction of the novel triazine sulfonamide analogues with various secondary amines and anilines generated various substituted triazine sulfonamide analogues of promising broad-spectrum activities including anti-microbial, anti-tumor, and anti-viral properties. The in vitro anti-proliferative activities of most of the novel compounds were evaluated on the NCI-60 cell line panel. The antifungal and antibacterial activities of the compounds were also estimated. The anti-viral activity against SARS CoV-2 virus was performed using MTT cytotoxicity assay to evaluate the half-maximal cytotoxic concentration (CC_50_) and inhibitory concentration 50 (IC_50_) of a representative compound from the novel triazine sulfonamide category. Compound **3a** demonstrated potent antiviral activity against SARS-CoV-2 with IC_50_ = 2.378 µM as compared to the activity of the antiviral drug remdesivir (IC_50_ = 10.11 µM). Our results indicate that, upon optimization, these new triazine sulfonamides could potentially serve as novel antiviral drugs.

## Introduction

Numerous antibiotics and other antimicrobials have been developed. However, the threat raised by antimicrobial resistance (AMR) is more recent and requires immediate attention [1, 2]. A significant increase in antibiotic resistance have been observed on a global level in the recent years. Almost seventeen million people die every year from infectious diseases, especially bacterial infections [3]. Many commercially available antibiotics are considered to be ineffective for treating microorganisms that have developed resistance to them [4]. Antibiotic resistance is a problem that has been related to antibiotic overuse, abuse, and a lack of new efficient drugs. Bacteria are considered major, urgent, and alarming concerns by the Centers for Disease Control and Prevention (CDC), many of which have a significant clinical and economic impact on the global population [5]. Due to the rapid increase in resistance to currently accessible commercially available antibiotics, it is imperative to develop novel antibacterial treatments with increased action to combat drug-resistant conditions [6].

In order to address drug resistance concerns and to treat opportunistic microbial infections, researchers have reported triazine core molecules displaying high antimicrobial potency in terms of antifungal and antibacterial. To fight against human disease-causing pathogens some studies were reported to synthesize different triazines [7–9] such as thiazole-triazines [10, 11], quinoline-triazine core [12, 13], quinazoline–triazine derivatives [14], coumarinyl-triazine derivatives [15], fullerene-based triazine compounds [16], disubstituted-s-triazines [17], tri-substituted s-triazine [18], s-triazine nucleobases [19], and many other s-triazine derivatives [20, 21].

Its worthy to note that there are many naturally occurring and synthetic potent compounds that comprise the triazine ring [22]. The triazine ring system constitutes one of the most promising scaffolds for drug discovery [23–27]. Triazine derivatives are biologically potent compounds with inhibitory activity towards tubulin [28], metalloproteinases [29], histone deacetylases [30], urease and tyrosinase [31]. Additionally, some of them inhibit protein kinases involved in critical signaling pathways that promote cancer cell proliferation, comprising glycogen synthase kinase 3 [32], cyclin-dependent kinases [25], ABL kinase [33], and casein kinase 2 [34]. The range of potential molecular targets for these compounds was expanded by the addition of the sulfonamide scaffold to the triazine derivatives [35, 36]. The sulfonamide moiety has attracted a lot of interest in medicinal chemistry, as a number of sulfonamides have been synthesized with a varied range of biological activities, including anti-fungal, anti-bacterial, anti-oxidant, anti-diabetic, anti-inflammatory [37], and anti-cancer potencies [37–44]. The FDA has approved various sulfonamide derivatives for use in cancer therapy [37]. Moreover, sulfonamides are considered as effective compounds possessing inhibitory effect on CAs. Ethoxzolamide, acetazolamide, methazolamide and dorzolamide are sulfonamide drugs utilized clinically in treating various pathological conditions [45–47].

**Fig. 1:**
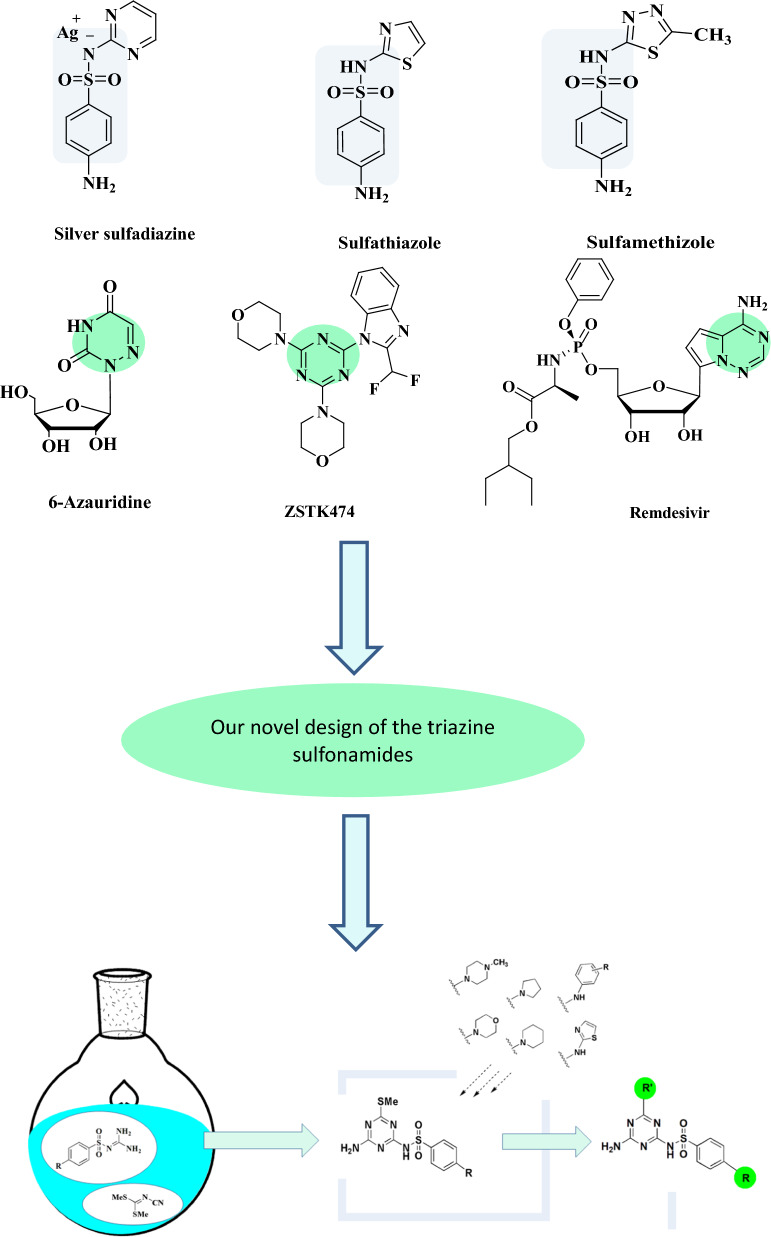
Potent sulfonamides & Triazines and our novel design of the triazine sulfonamides

In addition, sulfonamides are known to be effective as antimicrobial drugs such as silver sulfadiazine drug (Fig. 1) *(Flamazine, Silvadene, Ssd, Thermazene)* which is considered as a topical sulfonamide antibiotic that acts on the bacterial cell wall and cell membrane; approved for treating burns [48]. Another example, sulfathiazole (Fig. 1) which is a short-acting sulfa drug was a widely used oral and topical antimicrobial until less toxic alternatives were discovered. The use of it is still sporadic, occasionally in combination with sulfacetamide and sulfabenzamide [49]. Another sulfonamide antibiotic called sulfamethizole (Brand Name: *Urobiotic*) (Fig. 1) also is used to treat a wide range of susceptible bacterial infections [50]. Furthermore, sulfonamides have anti-viral characteristics that can be utilized to develop drugs against enteroviruses, coxsackievirus B, encephalomyocarditis viruses, human parainfluenza viruses, adenoviruses, Ebola virus, HIV, Marburg virus, SARS-CoV-2 among other viruses [51].

Herein, we have synthesized novel triazines sulfonamides utilizing dimethyl *N*-cyanodithioiminocarbonate which is considered as an important compound used in the synthesis of various biologically active heterocycles [52, 53], noteworthy, we have previously used this active reagents in synthesizing many novel antimetabolite analogues [54–59].

## Results and discussion

### Chemistry

**Scheme 1.**
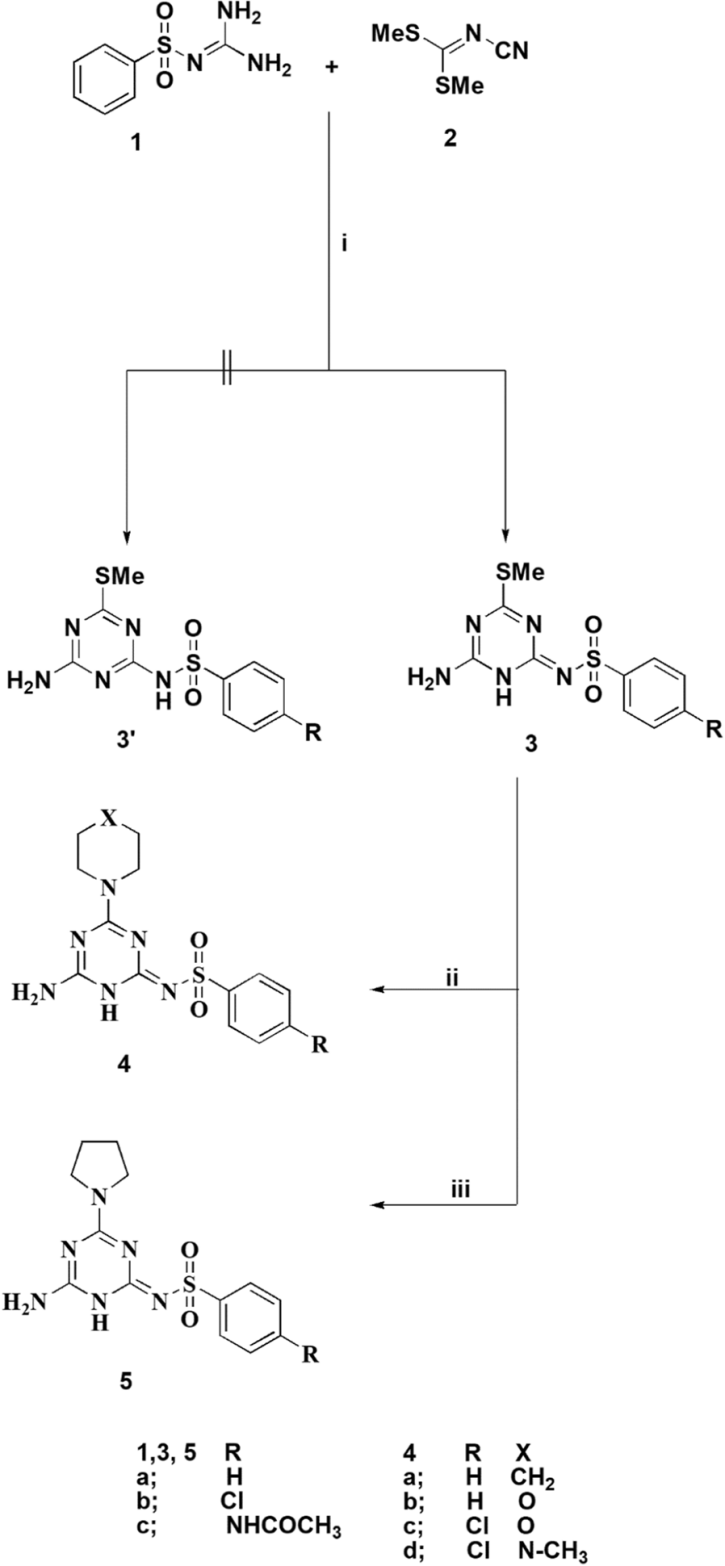
Reagents and conditions:** i** Dioxane, potassium hydroxide, reflux, 2 h. **ii** Dioxane, amine, potassium carbonate, reflux, 2 h. **iii** Dioxane, pyrrolidine, potassium carbonate, reflux, 2 h

**Fig. 2:**
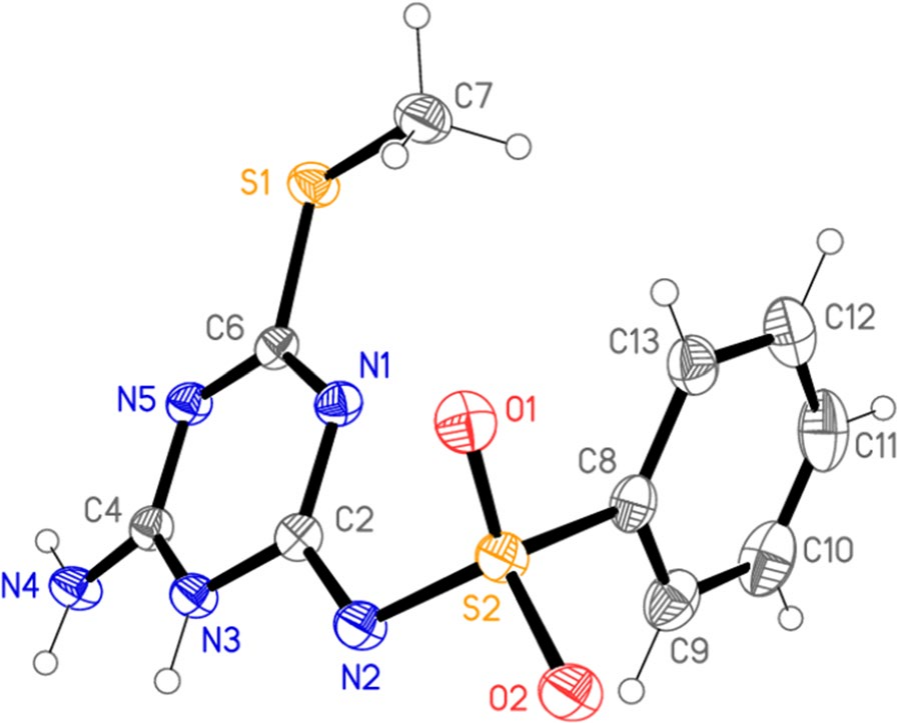
The molecule of Structure **3a** in the crystal. “The figure is reproduced via permission of the International Union of Crystallography under the open-access licence” [60]

The reaction of the substituted sulfaguanidine **1** with *N*-cyanodithioiminocarbonate **2** furnished the novel analogues of the triazine sulfonamide **3**. Cyclization of arylsulfonyl guanidine **1a–d** with compound **2** occurs in the presence of potassium hydroxide in dioxane under reflux to afford the targeted products **3a–c** (Scheme 1). The ^1^H NMR spectrum of compound **3a** revealed the presence of a singlet signal δ 2.29 ppm for the three protons of the methythio group, singlet at δ 7.35 for the NH_2_ Protons, and in the range from δ 7.54 to δ 7.98 ppm the aromatic protons were detectable, additionally the NH protons was appeared at δ 11.83 ppm. The structure of the compound is confirmed via single X-ray diffraction analysis as depicted in Fig. 2 [60].

**Scheme 2.**
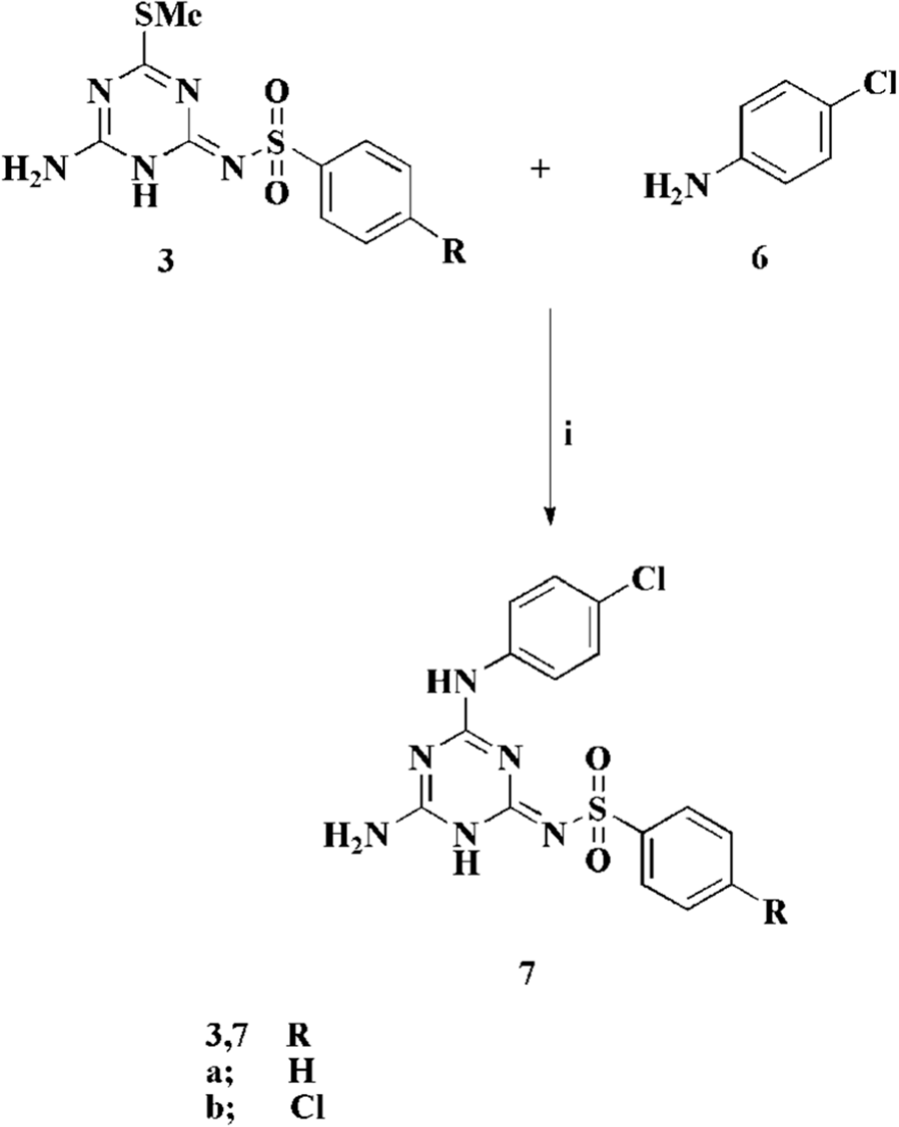
Reagents and conditions: **i** Dioxane, potassium carbonate, reflux, 2 h

Further reaction of the latter compounds with sec. amines such as morpholine, piperidine, *N*-methyl piperazine in the presence of potassium carbonate in refluxing dioxane furnishes the substituted triazine sulfonamides **4**. Additionally, the reaction of compound **3** with pyrrolidine generated compound **5**. The desired compounds were characterized using spectral and elemental analysis. The ^1^H NMR spectrum of compound **5a** revealed the presence of four multiplet signals at δ 1.76. ppm, two multiptet signals at δ 3.2 ppm, and two multiplet signals at δ 3.34 ppm of the methylene groups of the pyrrolidine moiety. Owing to the NH_2_ signal, it was appeared at δ 6.82 ppm, also the aromatic signals appeared at the range from δ 7.43 to δ 7.87 ppm, and the NH proton of the sulfonamide group was appeared at δ 11.19 ppm. In order to investigate the scope of this approach the triazine sulfonamides was reacted with aniline derivatives to afford a general methodology to the substituted triazine sulfonamides **7** (Scheme 2).

### Biological activity

#### In-vitro anti-proliferative activity

Estimation of in vitro antiproliferative activity was performed on the NCI-60 cell line panel. The US NIH's National Cancer Institute ("NCI") has selected the majority of structures for its Developmental Therapeutic Program (DTP). Various human tumor cell lines are used in screening procedures, including cell lines expressing brain, melanoma, leukemia, lung, ovarian, colon, kidney, prostate and breast malignancies.

The NCI screening process favors compounds with drug-like mechanisms of action according to computer-aided design. Whether the submitted compounds can diversify the NCI collection of small molecule compounds will govern which ones will be utilized for subsequent screening.

The compounds that have been elected were tested on the NCI cell panel and assumed the consistent NCI codes NSC D-840972, NSC D-840973, NSC D-D-840979, NSC D-840978, NSC D-840977, NSC D-840975 & NSC D-840976 to signify the diverse structures of this research. For each compound on each NCI cell line, the effects are expressed as a percentage of cell growth.

The lowest cell growth promotion for compound **3a** was against leukemia RPMI-8226 cell line (GP = 104.02%), non-small cell lung cancer EKVX cell line (GP = 77.46%), colon cancer HCT-15 cell line (GP = 96.34%), CNS cancer SNB-75 (GP = 74.76%), melanoma SK-MEL-5 (GP = 72.98%), ovarian cancer OVCAR-4 (GP = 95.65%), renal cancer CAKI-1 (GP = 88.61%), prostate cancer PC-3 (GP = 103.05%), and breast cancer MCF7 (GP = 84.02%). Compound **3b** displayed the lowest cell growth promotion against leukemia HL-60(TB) cell line (GP = 90.58%), non-small cell lung cancer EKVX cell line (GP = 80.79%), colon cancer HCT-15 cell line (GP = 97.17%), CNS cancer SNB-75 (GP = 77.59%), melanoma MALME-3 M (GP = 84.52%), ovarian cancer OVCAR-4 (GP = 91.13%), renal cancer CAKI-1 (GP = 81.13%), prostate cancer DU-145 (GP = 107.83%), and breast cancer MCF7 (GP = 87.47%).

Additionally, the lowest cell growth promotion for compound **4a** was against leukemia K-562 cell line (GP = 98.62%), non-small cell lung cancer HOP-92 cell line (GP = 89.70%), colon cancer HCT-15 cell line (GP = 97.79%), CNS cancer SNB-75 (GP = 77.71%), melanoma SK-MEL-5 (GP = 92.87%), ovarian cancer OVCAR-4 (GP = 86.36%), renal cancer UO-31 (GP = 85.43%), prostate cancer PC-3 (GP = 106.53%), and breast cancer MCF7 (GP = 93.45%). Alongside compound **4b** showed the lowest cell growth promotion against leukemia SR cell line (GP = 87.88%), non-small cell lung cancer EKVX cell line (GP = 90.00%), colon cancer HCT-15 cell line (GP = 99.61%), CNS Cancer SNB-75 (GP = 78.73%), melanoma UACC-62 (GP = 94.30%), ovarian cancer OVCAR-4 (GP = 90.71%), renal cancer CAKI-1 (GP = 89.24%), prostate cancer DU-145 (GP = 113.40%), and breast cancer MCF7 (GP = 91.47%).

The lowest cell growth promotion for compound **5a** was against leukemia SR cell line (GP = 90.10%), non-small cell lung cancer EKVX cell line (GP = 80.80%), colon cancer HCT-116 cell line (GP = 98.89%), CNS cancer SNB-75 (GP = 75.30%), melanoma UACC-62 (GP = 80.87%), ovarian cancer OVCAR-4 (GP = 83.44%), renal cancer UO-31 (GP = 81.59%), prostate cancer PC-3 (GP = 91.32%), and breast cancer MDA-MB-231/ATCC (GP = 88.39%). Meanwhile, compound **5b** exhibited the lowest cell growth promotion against leukemia HL-60 (TB) cell line (GP = 93.23%), non-small cell lung cancer EKVX cell line (GP = 80.41%), colon cancer HCT-116 cell line (GP = 95.84%), CNS cancer SNB-75 (GP = 77.45%), melanoma UACC-62 (GP = 87.29%), ovarian cancer OVCAR-4 (GP = 85.99%), renal cancer CAKI-1 (GP = 86.60%), prostate cancer DU-145 (GP = 105.36%), and breast cancer MCF7 (GP = 86.42%).

Furthermore the lowest cell growth promotion for compound **7b** was against leukemia HL-60 (TB) cell line (GP = 78.31%), non-small cell lung cancer EKVX cell line (GP = 92.73%), colon cancer HCT-15 cell line (GP = 98.79%), CNS cancer SNB-75 (GP = 81.18%), melanoma SK-MEL-5 (GP = 87.34%), ovarian cancer OVCAR-4 (GP = 94.63%), renal cancer CAKI-1 (GP = 87.08%), prostate cancer DU-145 (GP = 108.61%), and breast cancer MCF7 (GP = 97.13%).

In conclusion it is remarkable that compound **3a** the most potent among the estimated compounds, revealed remarkably lowest cell growth promotion against melanoma SK-MEL-5 (GP = 72.98%), CNS cancer SNB-75 (GP = 74.76%), and non-small cell lung cancer EKVX cell line (GP = 77.46%). Compound **7b** showed the lowest cell growth promotion against leukemia HL-60(TB) cell line (GP = 78.31%), **3b** renal cancer CAKI-1 (GP = 81.13%). Compound **5a** revealed the lowest cell growth promotion against ovarian cancer OVCAR-4 (GP = 83.44%), prostate cancer PC-3 (GP = 91.32%), and breast cancer MDA-MB-231/ATCC (GP = 88.39%). Additionally, compound **5b** showed the lowest cell growth promotion against colon cancer HCT-116 cell line (GP = 95.84%) (Table 1).

**Table 1:** Anti-tumor properties of the compounds at a dose of 10 μM using human tumor cell lines

Panel/Cell line	**3a**	**3b**	**4a**	**4b**	**5a**	**5b**	**7b**
*Leukemia*							
CCRF-CEM	109.88	100.29	116.66	106.84	96.89	116.52	116.17
HL-60(TB)	112.07	90.58	114.70	114.78	109.12	93.23	78.31
K-562	104.72	91.75	98.62	97.56	92.02	95.72	88.84
MOLT-4	108.90	99.31	98.73	103.63	107.38	99.53	95.99
RPMI-8226	104.02	105.57	100.08	113.68	92.47	99.37	107.97
SR	117.75	98.34	105.41	87.88	90.10	106.56	103.56
*Non-small cell lung cancer *							
A549/ATCC	98.66	98.81	105.02	105.10	105.57	105.50	103.59
EKVX	77.46	80.79	90.29	90.00	80.80	80.41	92.73
HOP-62	85.52	93.97	95.69	98.70	96.56	97.94	102.04
HOP-92	122.02	129.24	89.70	117.23	96.50	91.06	118.08
NCI-H226	93.04	94.32	94.39	98.66	86.85	95.95	96.79
NCI-H23	103.76	94.93	96.92	102.47	92.18	98.55	105.42
NCI-H322M	101.09	98.60	102.91	96.07	99.63	97.47	104.87
NCI-H460	103.67	103.16	103.61	102.31	100.92	100.37	102.22
NCI-H522	98.70	86.83	98.57	93.50	85.01	93.04	94.94
*Colon cancer*							
COLO 205	109.77	117.05	107.26	108.68	104.06	109.34	109.93
HCC-2998	108.67	120.78	110.46	108.19	103.14	110.53	111.51
HCT-116	99.60	100.28	99.11	101.85	98.89	95.84	102.90
HCT-15	96.34	97.17	97.79	99.61	101.80	100.44	98.79
HT29	108.29	116.29	105.23	102.68	104.11	109.46	112.65
KM12	106.63	106.43	109.05	104.54	102.17	101.19	104.16
SW-620	105.70	100.74	101.32	106.70	99.76	98.98	105.00
*CNS cancer*							
SF-268	96.54	98.96	101.29	103.77	95.21	96.49	99.36
SF-295	90.83	86.82	93.19	93.07	86.80	90.82	97.23
SF-539	101.08	92.86	97.24	97.97	89.91	95.16	92.58
SNB-19	98.77	95.13	95.81	95.83	90.09	97.19	95.53
SNB-75	74.76	77.59	77.71	78.73	75.30	77.45	81.18
U251	103.95	99.30	104.64	107.53	97.45	107.68	99.84
*Melanoma*							
LOX IMVI	95.77	94.66	98.93	97.31	89.29	97.71	94.31
MALME-3 M	97.44	84.52	101.06	100.69	93.92	91.01	102.01
M14	102.37	101.94	104.95	98.91	101.13	100.91	99.60
MDA-MB-435	100.60	101.75	98.30	98.72	99.57	100.87	99.48
SK-MEL-2	128.41	102.83	112.24	105.73	116.13	110.65	111.32
SK-MEL-28	99.39	101.04	103.77	101.19	96.01	95.97	97.92
SK-MEL-5	72.98	106.78	92.87	95.62	85.03	95.30	87.34
UACC-257	99.97	103.60	102.15	103.01	111.28	105.19	105.56
UACC-62	103.11	84.71	94.57	94.30	80.87	87.29	94.38
*Ovarian cancer*							
IGROV1	103.94	97.31	110.12	106.47	100.49	89.71	107.05
OVCAR-3	114.38	110.36	106.76	112.06	105.84	106.71	108.62
OVCAR-4	95.65	91.13	86.36	90.71	83.44	85.99	94.63
OVCAR-5	104.49	100.03	103.05	100.77	96.50	91.37	99.69
OVCAR-8	104.22	101.58	104.63	106.02	103.10	101.58	105.11
NCI/ADR-RES	100.60	99.63	103.92	102.27	99.25	101.70	100.28
SK-OV-3	103.08	91.32	107.02	113.01	93.62	98.21	101.24
*Renal cancer*							
786-0	103.15	108.31	110.30	102.34	100.37	101.72	99.30
A498	101.04	110.05	115.02	105.58	103.47	112.37	98.23
ACHN	99.94	98.49	102.14	101.60	91.57	101.12	102.62
CAKI-1	88.61	81.13	92.13	89.24	83.53	86.60	87.08
RXF 393	118.89	105.80	102.59	101.71	90.00	105.38	104.55
SN12C	101.86	96.10	96.05	98.77	85.63	95.34	100.37
TK-10	101.71	134.03	104.72	93.71	107.73	125.56	104.11
UO-31	92.40	81.52	85.43	89.82	81.59	89.98	92.55
*Prostate cancer*							
PC-3	103.05	112.09	106.53	113.83	91.32	107.65	115.88
DU-145	106.44	107.83	109.84	113.40	109.35	105.36	108.61
*Breast cancer*							
MCF7	84.02	87.47	93.45	91.47	90.37	86.42	97.13
MDA-MB-231/ATCC	98.98	90.59	100.13	93.08	88.39	92.68	104.60
HS 578T	99.34	97.30	107.63	113.10	93.30	99.61	111.99
BT-549	104.79	106.72	110.89	109.75	99.59	98.36	105.65
T-47D	94.87	92.07	93.59	98.98	91.58	95.54	99.03

#### Antimicrobial evaluation

Most of the novel compounds were estimated for their in vitro anti-bacterial efficacy against some species of Gram (− ve) bacteria, namely, *Escherichia coli, Klebsiella pneumonia*, and *Pseudomonas aeruginosa,* along with two Gram (+ ve) bacteria, namely, *Staphylococcus aureus* and *Streptococcus mutans.* Additionally, their effectiveness against the fungus *Candida albicans* was assessed. To estimate the preliminary anti-bacterial and anti-fungal potencies, the agar-diffusion method was utilized.

Nystatin, Ampicillin, and Gentamicin were also used as standard drugs against fungal, Gram + ve bacterial, and Gram − ve bacterial strains, respectively. The reports of the antimicrobial results were expressed as the average diameter of inhibition zones of the microbial growth around the disks in mm values, as accomplished in Table 2. The optimization of antimicrobial evaluation was performed utilizing a statistical experimental design [61–63].

**Table 2:** Determination of the antimicrobial activity of compounds (**3a**, **3b, 3c**, **4a**, **4b** and **4c**) against different antibacterial and fungal strains

Microorganism	Sample						Standard antibiotic
	**3a**	**3b**	**3c**	**4a**	**4b**	**4c**	
**Gram negative bacteria**							Gentamicin
*Escherichia coli* (ATCC:10536)	NA*	NA	NA	11.6 ± 0.6	NT	NA	27 ± 1.0
*Klebsiella pneumonia* (ATCC:10031)	NA	NT**	NA	NT	NT	NA	25 ± 1.0
*Pseudomonas aeruginosa* (ATCC:27853)	NA	NT	NA	NT	NT	NA	27.3 ± 0.6
**Gram positive bacteria**							Ampicillin
*Staphylococcus aureus* (ATCC:13565)	NA	NA	NA	11.3 ± 0.6	NA	NA	21.7 ± 0.6
*Streptococcus mutans* (ATCC:25175)	NA	NA	NA	NA	NA	NA	30 ± 1.0
**Fungi**							Nystatin
*Candida albicans* (ATCC:10231)	12.3 ± 0.6	NA	13.3 ± 0.6	NA	NA	NA	21 ± 1.0

*NA: No activity; **NT: Not tested

**Fig. 3:**
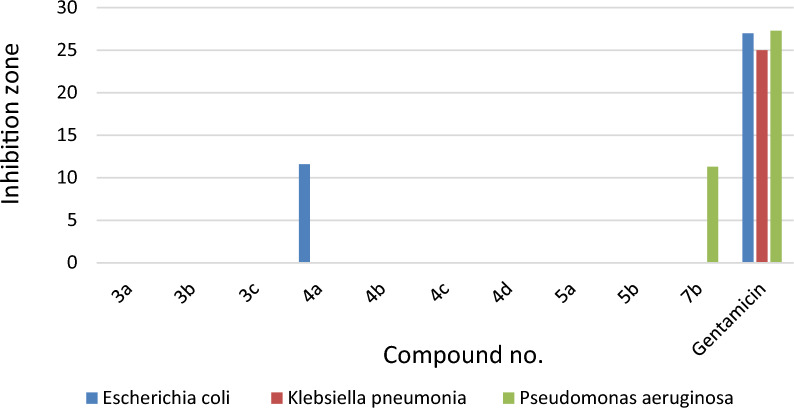
The antibacterial activities of compounds **3a-c**, **4a-d**, **5a,b**, and **7b** as compared with Gentamicin as standard antibiotic against Gram (–ve) bacteria

**Fig. 4:**
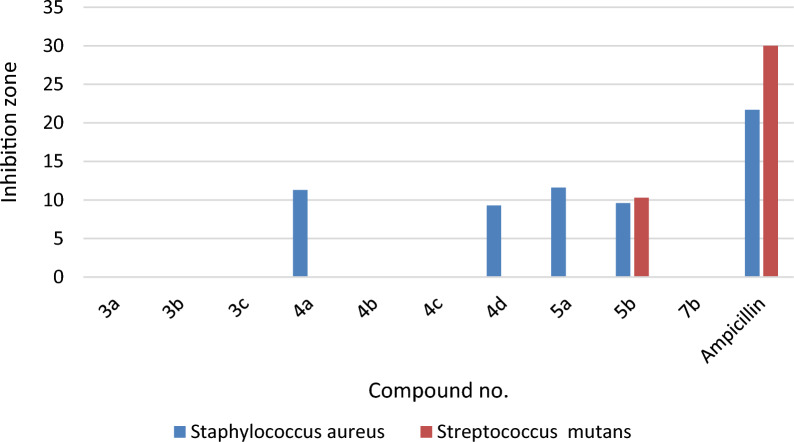
The antibacterial activities of compounds **3a-c**, **4a-d**, **5a**,**b**, and **7b** as compared with ampicillin as standard antibiotic against Gram (+ ve) bacteria

**Fig. 5:**
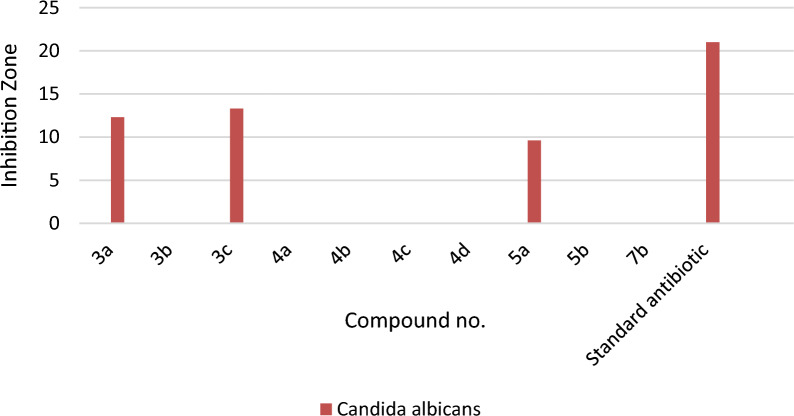
The antifungal activities of compounds **3a-c**, **4a-d**, **5a**,**b**, and **7b** as compared with Nystatin as standard antibiotics against Candida albicans

As depicted in Table 2 and in Figs. 3, 4, 5, compound **4a** showed some activities against the gram negative bacterial strain, *Escherichia coli* (ATCC:10,536) (inhibition zone 11.6 ± 0.6 mm( compared to Gentamicin (inhibition zone 27 ± 1.0 mm), while revealing inhibition zone against the gram positive strain, *Staphylococcus aureus* (ATCC:13,565) (inhibition zone 11.3 ± 0.6 mm)*,* when compared to Ampicillin (inhibition zone 21.7 ± 0.6 mm). Its worthy to note that no apparent potency was observed for compound **4a** against the fungal strain *Candida albicans* (ATCC:10,231) compared to Nystatin (inhibition zone 21 ± 1.0 mm). On the other hand compound **3c** showed fungal zone of inhibition with the value 13.3 ± 0.6 mm against the *Candida albicans* (ATCC:10,231) compared to Nystatin (inhibition zone 21 ± 1.0 mm). Thus, **3c** is considered as the most potent compound with antifungal activity among the other tested compounds. Compound **5a** indicated inhibition zone against the gram-positive strain, *Staphylococcus aureus* (ATCC:13565) (inhibition zone 11.6 ± 0.6 mm)*.*

Compound **3a** and **5a** revealed fungal zone of inhibition with the value 12.3 ± 0.6 mm, and 9.6 ± 0.6, respectively against the *Candida albicans* (ATCC:10231) compared to Nystatin. Compound **7b** is considered is the only compound among the examined ones which revealed potency against the *Pseudomonas aeruginosa* (ATCC:27853) with inhibition zone value of 11.3 ± 0.6 respectively.

Against gram positive bacteria *Staphylococcus aureus* (ATCC:13565) compound **4d** indicated bacterial zone of inhibition with the value of 9.3 ± 0.6 mm. The activity of the **5b** against the gram-positive bacteria *Staphylococcus aureus,* and *Streptococcus mutans* with inhibition zone value of 9.6 ± 0.6, and 10.3 ± 0.6 respectively (Table 3). All the tested compounds revealed no apparent potency against *Klebsiella pneumonia* (ATCC:10031)*.*

**Table 3:** Determination of the antimicrobial activity of compounds (**4d**, **5a, 5b**, and **7b**) against different antibacterial and fungal strains

Microorganism	Sample				Standard antibiotic
	**4d**	**5a**	**5b**	**7b**	
**Gram negative bacteria**					Gentamicin
*Escherichia coli* (ATCC:10536)	NT	NA	NA	NA	27 ± 1.0
*Klebsiella pneumonia* *(ATCC:10031)*	NT	NA	NA	NA	25 ± 1.0
*Pseudomonas aeruginosa* (ATCC:27853)	NT	NA	NT	11.3 ± 0.6	27.3 ± 0.6
**Gram positive bacteria**					Ampicillin
*Staphylococcus aureus* (ATCC:13565)	9.3 ± 0.6	11.6 ± 0.6	9.6 ± 0.6	NA	21.7 ± 0.6
*Streptococcus mutans* (ATCC:25175)	NA	NA	10.3 ± 0.6	NA	30 ± 1.0
**Fungi**					Nystatin
*Candida albicans* (ATCC:10231)	NA	9.6 ± 0.6	NA	NA	21 ± 1.0

#### SARS-CoV-2

**Fig. 6:**
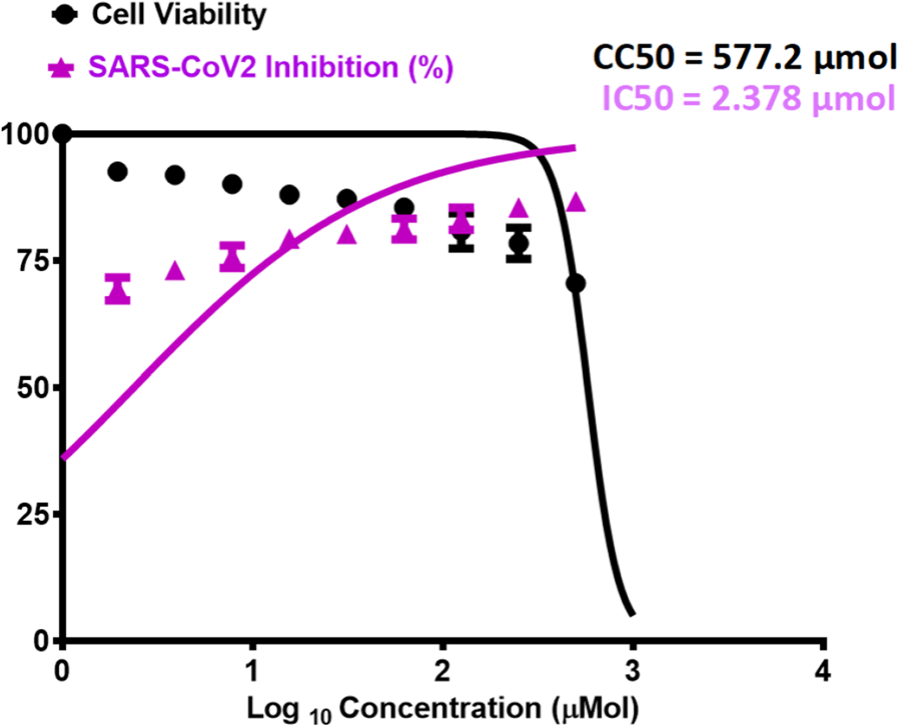
Graph of inhibitory concentration 50 (IC_50_) of tested compound **3a**: Antiviral activity against SARS-CoV-2

The novel synthesized compound **3a** was evaluated for its anti-viral potency against SARS CoV-2 virus to determine the half-maximal cytotoxic concentration (CC_50_) and inhibitory concentration 50 (IC_50_) (Fig. 6). The antiviral activity of the compound is identified using the MTT assay. The results revealed that compound **3a** has high and potent antiviral activity against SARS-CoV-2.

**Fig. 7:**
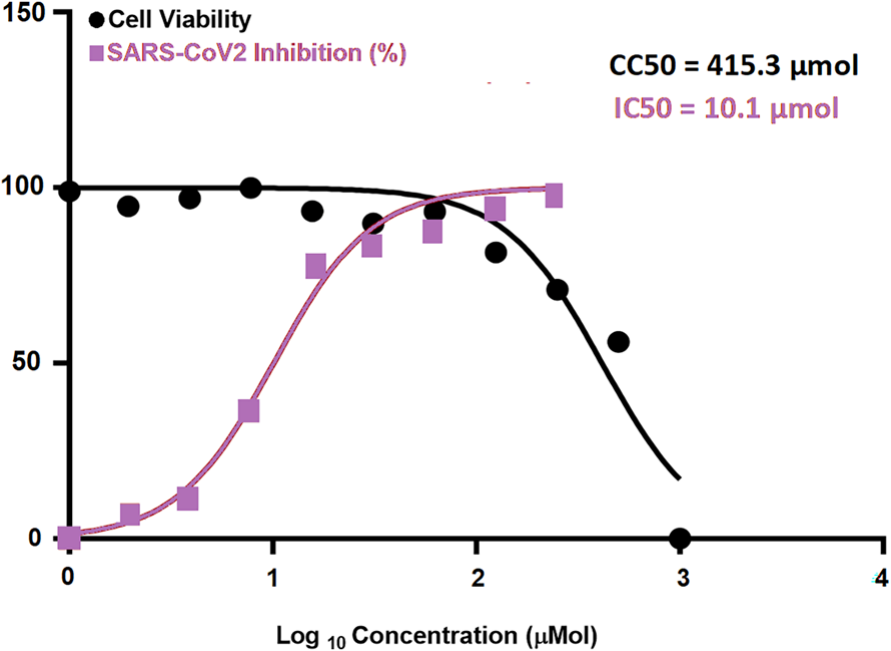
Graph of inhibitory concentration 50 (IC_50_) of remdesivir as positive control. Antiviral activity against SARS-CoV-2

The inhibition concentration (IC50) was calculated from the slope on graph pad prism for compound **3a** and according to that value, the promising compound had low value can inhibit propagation of virus in the same time with low toxicity on the cell as compound **3a** had IC50 = 2.378 µM and CC50 = 577.2 µM with safety index = 250. Thus, compound **3a** showed potent antiviral activity against SARS-CoV-2 with IC_50_ = 2.378 µM that is comparable to the activity of the antiviral drug remdesivir (IC_50_ = 10.11 µM) (Fig. 7). Compound **3a** revealed a selectivity index (SI = (CC_50_/IC_50_) = 250) that is much higher than the selectivity index of remdesivir as positive control (SI = 41.07).

Cytotoxicity assay of compound **3a** in Vero E6 cells is shown in Fig. 3. The determination of the cytotoxicity of compound **3a** and remdesivir based on the dose response was performed utilizing MTT assay. The calculations of the 50% cytotoxic concentration (CC_50_) for the compound is identified via non-linear regression analysis of GraphPad Prism software (version 5.01). The inhibitory concentration 50% (IC_50_) values were also calculated utilizing non-linear regression analysis of GraphPad Prism software through plotting log inhibitor versus the normalized response known as the variable slope.

## Conclusion

In conclusion, the synthesis of triazine sulfonamides and its analogues were achieved starting from sulfaguanidine derivatives. Our synthetic approach is expected to contribute in the provision of a wide range of triazine sulfonamide analogs starting from the crucial intermediate *N*-cyanodithioiminocarbonate. The insertion of several amines or aryl groups yielded the novel substituted triazine sulfonamides. The in vitro anti-proliferative activities, the antimicrobial activities and the antiviral activity against SARS-CoV-2 virus were evaluated. Compounds **4a**, **4d** & **5b** showed some activities against the gram (–ve) and gram (+ ve) bacterial strains compared to Gentamicin and Ampicillin. Compounds **3a**, **3c** and **5a** displayed potency against the fungal strain *Candida albicans* compared to Nystatin as standard anti-fungal drug. The anti-proliferative efficacy of the novel triazine sulfonamides was also estimated on NCI 60 cancer cell lines. Compound **3a** is considered to be the most potent derivative among the estimated compounds in which it revealed remarkably lowest cell growth promotion against melanoma SK-MEL-5, CNS cancer SNB-75, and non-small cell lung cancer EKVX cell line. Additionally, the anti-viral activity against SARS CoV-2 virus was performed utilizing the MTT cytotoxicity assay. Compound **3a** exhibited antiviral potency against SARS-CoV-2 with IC_50_ = 2.378 µM as compared to the antiviral drug remdesivir (IC_50_ = 10.11 µM). This study showed promising results for developing these novel structures. Further studies concerning synthesizing other triazine sulfonamide analogs and the evaluation of their biological potency are currently in progress.

## Experimental

### Methods

On the pre-coated silica gel 60 F245 aluminum plates, TLC was utilized for monitoring the reaction's development and the UV light was used for visualization. The Stuart SMP30 equipment was used to determine the melting point that was uncorrected. In the faculty of Pharmacy at the Drug Discovery, Research & Development Centre at Ain Shams University and in the National Research Center, Egypt, the spectroscopic analyses of the compounds were carried out. On Bruker Fourier 400 and 500 (operating at 400 MHz and 500 MHz, respectively) at 300 K, the NMR spectra were measured. The National Cancer Institute in Bethesda, Maryland, United States, conducted the anticancer screening. Antimicrobial evaluation carried out at the Cairo University's Faculty of Science's Microbiology Unit in the Biochemistry Central Lab, Cairo, Egypt. The Centre of Scientific Excellence for Influenza Viruses, National Research Centre (NRC), Dokki, Cairo 12622, Egypt, conducted the antiviral assays for the SARS-CoV-2 virus.

### Synthesis

#### *Synthesis of N*-[3,4-dihydro-4-amino-6-(methylthiol)triazin-2-yl]benzenesulfonamide derivatives 3

**General procedure I**: A mixture of substituted sulfaguanidine **1** (0.01 mol) with *N*-cyanodithioiminocarbonate** 2** (0.01 mol) in dry dioxane (20 mL) containing potassium hydroxide (0.015 mol) was refluxed for 3 h. The reaction mixture was poured into ice-water, filtered, washed thoroughly with water, dried and crystallized from ethanol to obtain the desired product.

##### Synthesis of N-[3,4-dihydro-4-amino-6-(methylthiol)triazin-2-yl]benzenesulfonamide (3a)

According to general procedure I, compound **2** reacted with benzenesulfonylguanidine (**1a**) to afford compound **3a** as an off white solid (87%); mp 247–249 °C; IR (KBr, cm^−1^): υ 3261, 3202 (NH), 3065 (Ar–CH), 2931, 2813 (alph. CH), 1555 (C═C), 1358, 1141 (SO_2_); ^1^H NMR (400 MHz, DMSO-*d*_*6*_): δ 2.29 (s, 3H, CH_3_), 7.35 (s, 2H, NH_2_), 7.54–7.64 (m, 3H, Ar–H), 7.96–7.98 (d, 2H, Ar–H), 11.83 (s, 1H, NH); ^13^C-NMR (400 MHz, DMSO-*d*_6_) δ (ppm): 12.53,127.75, 128.75, 132.79, 140.85, 159.76, 163.42, 180.34. Anal. Calcd. For. C_10_H_11_N_5_O_2_S_2_ (297.36): C, 40.39; H, 3.73; N, 23.55; S, 21.57. Found: C, 40.38; H, 3.72; N, 23.55; S, 21.56.

##### *Synthesis of N*-[3,4-dihydro-4-amino-6-(methylthiol)triazin-2-yl]-4-chlorobenzenesulfonamide (3b)

According to general procedure I, compound **2** reacted with *p*-chlorobenzenesulfonylguanidine (**1b**) to afford compound **3b** as an off white solid (84%); mp 283–285 °C; IR (KBr, cm^−1^): υ 3455, 3260, 3201 (NH), 2980 (alph. CH), 1555 (C═C), 1359, 1141 (SO_2_); ^1^H NMR (400 MHz, DMSO-*d*_*6*_): δ 2.49 (s, 3H, CH_3_), 7.41 (s, 1H, NH), 7.60–7.62 (d, 2H, Ar–H), 7.74 (s, 1H, NH), 7.94–7.97 (d, 2H, Ar–H), 11.79 (s, 1H, NH); ^13^C-NMR (400 MHz, DMSO-* d*_6_) δ (ppm): 12.50, 128.70, 129.70, 137.33, 140.05. Anal. Calcd. For. C_10_H_10_ClN_5_O_2_S_2_ (331.8): C, 36.20; H, 3.04; Cl, 10.68; N, 21.11; S, 19.33. Found: C, 36.20; H, 3.03; Cl, 10.67; N, 21.11; S, 19.32.

##### *Synthesis of N*-[3,4-dihydro-4-amino-6-(methylthiol)triazin-2-yl]-4-acetamidobenzenesulfonamide (3c)

According to general procedure I, compound **2** reacted with *p*-acetamidobenzenesulfonylguanidine (**1c**) to afford compound **3c** as a brown solid (40%); mp ˃340 °C; ^1^H NMR (400 MHz, DMSO-*d*_*6*_): δ ^1^H NMR (400 MHz, DMSO-*d*_*6*_): δ 2.41 (s, 3H, CH_3_), 2.49 (s, 3H, CH_3_), 7.00 (s, 1H, NH), 7.26 (s, 1H, NH_2_), 7.27–7.29 (d, 2H, Ar–H), 7.51–7.53 (d, 2H, Ar–H), 7.91 (s, 1H, NH), 8.68 (s, 1H, NH); Anal. Calcd. For. C_12_H_14_N_6_O_3_S_2_ (354.41): C, 40.67; H, 3.98; N, 23.71; S, 18.09. Found: C, 40.67; H, 3.97; N, 23.70; S, 18.08.

#### Synthesis of substituted N-[3,4-dihydro-4-amino-6-(methylthiol)triazin-2-yl]benzenesulfonamides 4, 5, and 7

**General procedure II**: A mixture of *N*-[3,4-dihydro-4-amino-6-(methylthiol)triazin-2-yl]benzenesulfonamide (0.01 mol) with various secondary amines (0.02 mol), or anilines **7** (0.01 mol) in dry dioxane (20 mL) containing potassium carbonate (0.015 mol) was refluxed for 3 h. The reaction mixture was poured into ice-water, filtered, washed thoroughly with water, dried and crystallized from ethanol to obtain the desired product.

##### Synthesis of N-[3,4-dihydro-4-amino-6-(piperidin-1-yl)triazin-2-yl]benzenesulfonamide (4a)

According to general procedure II, compound **3** reacted with piperidine to afford compound **5a** as a buff solid (62%); mp 319–320 °C; ^1^H NMR (400 MHz, DMSO-*d*_*6*_): δ 1.30 (m, 2H, CH_2_), 2.49 (m, 4H, CH_2_), 3.62 (m, 4H, CH_2_), 6.13 (s, 2H, NH_2_), 7.31–7.35 (m, 1H, Ar–H), 7.37–7.44 (m, 2H, Ar–H), 7.76–7.78 (m, 1H, Ar–H), 7.86–7.89 (d, 1H, Ar–H), 9.53 (s, 1H, NH); Anal. Calcd. For. C_14_H_18_N_6_O_2_S (334.4): C, 50.28; H, 5.43; N, 25.13; S, 9.59. Found: C, 50.27; H, 5.43; N, 25.12; S, 9.58.

##### Synthesis of N-[3,4-dihydro-4-amino-6-morpholinotriazin-2-yl]benzenesulfonamide (4b)

According to general procedure II, compound **3a** reacted with morpholine to afford compound **4b** as an off white solid (73%); mp 360–363 °C; IR (KBr, cm^−1^): υ 2985(Ar–CH), 2972, 2907, 2873, 2850 (alph. CH), 1550 (C═C), 1357, 1135 (SO_2_); ^1^H NMR (400 MHz, DMSO-*d*_*6*_): δ 2.62 (m, 2H, CH_2_), 3.11 (m, 2H, CH_2_), 3.47 (m, 4H, CH_2_), 5.67 (s, 1H, NH), 6.09 (s, 2H, NH_2_), 7.31 (m, 3H, Ar–H), 7.70 (m, 1H, Ar–H), 7.76 (m, 1H, Ar–H); Anal. Calcd. For. C_13_H_16_N_6_O_3_S (336.37): C, 46.42; H, 4.79; N, 24.98; S, 9.53. Found: C, 46.42; H, 4.79; N, 24.97; S, 9.52.

##### Synthesis of N-[3,4-dihydro-4-amino-6-morpholinotriazin-2-yl]-4-chlorobenzenesulfonamide (4c)

According to general procedure II, compound **3b** reacted with morpholine to afford compound **4c** as off white solid (81%); mp 342–343 °C; Anal. Calcd. For. C_13_H_15_ClN_6_O_3_S (370.81): C, 42.11; H, 4.08; Cl, 9.56; N, 22.66; S, 8.65. Found: C, 42.11; H, 4.07; Cl, 9.55; N, 22.66; S, 8.64.

##### Synthesis of N-[3,4-dihydro-4-amino-6-(4-methylpiperazin-1-yl)-triazin-2-yl]-4-chlorobenzenesulfonamide (4d)

According to general procedure II, compound **3b** reacted with 4-methylpiperazine to afford compound **4d** as a buff solid (51%); mp 270–273 °C; Anal. Calcd. For. C_14_H_18_ClN_7_O_2_S (383.86): C, 43.81; H, 4.73; Cl, 9.24; N, 25.54; S, 8.35. Found: C, 43.81; H, 4.73; Cl, 9.23; N, 25.53; S, 8.34.

##### Synthesis of N-[3,4-dihydro-4-amino-6-(pyrrolidin-1-yl)triazin-2-yl]benzenesulfonamide (5a)

According to general procedure II, compound **3a** reacted with pyrrolidine to afford compound **5a** as buff crystals (81%); mp 317 °C; ^1^H NMR (400 MHz, DMSO-*d*_*6*_): δ 1.76–1.77 (m, 4H, CH_2_); 3.20–3.23 (m, 2H, CH_2_), 3.32–3.36 (m, 2H, CH_2_), 6.82 (s, 2H, NH_2_), 7.43–7.50 (m, 2H, Ar–H), 7.84–7.87 (d, 2H, Ar–H), 7.97–7.99 (d, 1H, Ar–H), 11.19 (s, 1H, NH); ^13^C-NMR (400 MHz, DMSO-* d*_6_) δ (ppm): 12.87, 24.95, 25.03, 46.80, 46.97, 127.66, 128.13, 129.11, 131.26, 144.42. Anal. Calcd. For. C_13_H_16_N_6_O_2_S (320.37): C, 48.74; H, 5.03; N, 26.23; S, 10.01. Found: C, 48.74; H, 5.02; N, 26.22; S, 10.00.

##### Synthesis of N-[3,4-dihydro-4-amino-6-(pyrrolidin-1-yl)triazin-2-yl]-4-chlorobenzenesulfonamide (5b)

According to general procedure II, compound **3b** reacted with pyrrolidine to afford compound **5b** as a buff solid (78%); mp 237 °C; IR (KBr, cm^−1^): υ 3346, 3209 (NH), 2971(Ar–CH), 2873 (alph. CH), 1541 (C═C), 1390, 1132 (SO_2_); ^1^H NMR (400 MHz, DMSO-*d*_*6*_): δ 1.76 (m, 2H, CH_2_); 1.82–1.85 (m, 2H, CH2), 3.13–3.21 (m, 4H, CH_2_), 5.79 (s, 1H, NH), 6.17 (s, 1H, NH_2_), 6.61 (s, 1H, NH), 7.34–7.40 (m, 2H, Ar–H), 7.49–7.52 (d, 1H, Ar–H), 7.77–7.80 (d, 1H, Ar–H); Anal. Calcd. For. C_13_H_15_ClN_6_O_2_S (354.82): C, 44.01; H, 4.26; Cl, 9.99; N, 23.69; S, 9.04. Found: C, 44.00; H, 4.25; Cl, 9.97; N, 23.67; S, 9.01.

##### Synthesis of N-[3,4-dihydro-4-amino-6-(N-(4-chlorophenyl))triazin-2-yl]benzenesulfonamide (7a)

According to general procedure II, compound **3a** reacted with 4-chloroaniline (**6**) to afford compound **7a** as a buff solid (68%); ^1^H NMR (400 MHz, DMSO-*d*_*6*_): δ δ 6.17 (s, 1H, NH), 6.57 (s, 1H, NH_2_), 7.34–7.39 (m, 4H, Ar–H), 7.49–7.51 (d, 2H, Ar–H), 7.77–7.80 (d, 3H, Ar–H), 8.54 (s, 1H, NH); Anal. Calcd. For. C_15_H_13_ClN_6_O_2_S (376.82): C, 47.81; H, 3.48; Cl, 9.41; N, 22.30; S, 8.51. Found: C, 47.81; H, 3.48; Cl, 9.40; N, 22.30; S, 8.50.

##### *Synthesis of N-[3,4-dihydro-4-amino-6-* (*N-(4-chlorophenyl)) triazin-2-yl]-4-chlorobenzenesulfonamide (7b)*

According to general procedure II, compound **3b** reacted with 4-chloroaniline (**6**) to afford compound **7b** as a buff solid (71%); mp ˃ 350 °C; IR (KBr, cm^−1^): υ 3160 (NH), 2934 (Ar–CH), 2619 (alph. CH), 1618 (C═C), 1364,1388, 1130 (SO_2_); ^1^H NMR (400 MHz, DMSO-*d*_*6*_): δ 6.17 (s, 1H, NH), 6.57 (s, 1H, NH), 7.74 (s, 1H, NH), 7.34–7.39 (m, 4H, Ar–H), 7.49–7.51 (d, 2H, Ar–H), 7.77–7.80 (d, 2H, Ar–H), 8.54 (s, 1H, NH_2_); Anal. Calcd. For. C_15_H_12_Cl_2_N_6_O_2_S (411.27): C, 43.81; H, 2.94; Cl, 17.24; N, 20.43; S, 7.80. Found: C, 43.80; H, 2.94; Cl, 17.23; N, 20.42; S, 7.78.

### In vitro anti-proliferative activity

Primary anticancer assays were carried out in accordance with NCI procedures [64–68]. The compounds were applied at a single concentration, and the cell culture was then incubated for 48 h. Sulforhodamine B (SRB), a protein-binding dye, was used to detect the endpoints. The compound's effects were displayed as a percentage growth (GP%) of the treated cells relative to the untreated cells in the control. The range of growth (%) displayed the maximum and lowest growth arising from the initial single high dosage (10^−5^M) sensitivity against the different cancer cell lines.

### Antimicrobial activity

Using the agar well diffusion method, the synthesized compounds were separately evaluated against a panel of Gram (+ ve) and Gram (−ve) bacterial pathogens and the fungi [69]. The compounds were evaluated against fungal and bacterial strains at a concentration of 15 mg/mL. In sterilized saline equivalent to 0.5 McFarland standard solution (1.5 × 10^5^ cfu/ml), the microbial suspension was prepared, then the turbidity of the medium was adjusted to the optical density (OD) = 0.13 at 625 nm utilizing a spectrophotometer. A sterile cotton swab should ideally be dipped into the adjusted suspension within fifteen minutes of adjusting the turbidity of the inoculum suspension, flooded over the dried agar surface, and then allowed to dry for another 15 min. Using a sterile borer, 6 mm-diameter wells were prepared in the solidified media. Using a micropipette, 100 μL of the tested compound solution was added to each well. At 37 °C, the plates were then incubated. Measuring the zone of inhibition (mm) was carried out after 24 h incubation at 30 °C for bacterial plates and 48 h for fugal plates. The results were recorded for each tested substance as % inhibition ± SD, and the experiment was run in triplicate. The inhibition zone s’ diameters were measured in millimeters.

### Cytotoxicity assay

#### SARS-CoV2

##### MTT cytotoxicity assay

To identify the half maximum cytotoxic concentration (CC_50_), stock solutions of the tested substances were prepared in DMSO (10% in ddH_2_O) and subsequently diluted to the employed concentrations using DMEM. By slightly altering the 3-(4,5-dimethylthiazol-2-yl)-2,5-diphenyltetrazolium bromide (MTT) technique, the cytotoxic activity of the extracts was examined in VERO-E6 cells. Briefly, the cells were seeded in 96-well plates at a density of 3 × 10^5^ cells per ml (100 µl /well) and then incubated for 24 h at 37 °C in 5% carbon dioxide.

After 24 h, the examined compounds were treated in triplicates to cells in a range of doses. The supernatant was removed twenty-four hours in advance, and cell mono-layers were then washed three times with sterile 1 × PBS before being incubated for four hours at 37 degrees Celsius with MTT solution (20 µl of a 5 mg/ml stock solution). The medium was then aspirated.

In each well, 200 µl of acidified isopropyl alcohol (0.04 M hydrochloric acid in isopropyl alcohol = 0.073 ml hydrochloric acid in 50 ml isopropyl alcohol) was used to dissolve the produced formazan crystals. Using a multiwall-plate reader, the absorbance of formazan solutions was calculated at max λ 540 nm and 620 nm. Using a plot of cytotoxicity versus sample concentration, the concentration (CC_50_) that indicated 50% cytotoxicity was determined.

##### Estimation of the inhibitory Concentration 50% (IC 50)

2.4 × 10^4^ Vero E6 cells were seeded onto tissue culture plates (96-well), and they were then exposed to 5% carbon dioxide at 37°C for the duration of the full night.

The cell monolayers were then treated with hCoV-19/Egypt/NRC-03/2020 (Accession No. on GSAID/ EPI ISL 430820) and allowed to remain there for an additional hour at ambient temperature. The cell monolayers were then covered with DMEM (100 μl) with various test drug doses.

The cells were then stained with 0.1% crystal violet in distilled water at ambient temperature for fifteen minutes, fixed with 100 μl polyoxymethylene (4%) for twenty minutes, and kept in a 5% carbon dioxide incubator at 37 °C for the ensuing 72 h. After being fixed with 100 μl polyoxymethylene (4%) for 20 min, the cells were stained with 0.1% crystal violet in DH_2_O at room temperature for 15 min. The crystal violet dye was then dissolved in 100 μl of methanol in each well (Anthos Labtec Instruments, Heerhugowaard, Netherlands) before the optical density of the color was determined at 570 nm using an Anthos Zenyth 200rt-plate reader. The amount of a chemical required to lower the virally-induced cytopathic effect (CPE) in contrast to virus control by 50% is known as the IC 50.

## Data Availability

The datasets generated during and/or analyzed during the current study are available from the corresponding author on reasonable request.

## References

[1] Fischbach MA, Walsh CT (2009). Antibiotics for emerging pathogens. Science.

[2] Zhu YG, Zhao Y, Li B, Huang C, Zhang S, Yu S, Chen Y, Zhang T, Gillings MR, Su J (2017). Continental-scale pollution of estuaries with antibiotic resistance genes. Nat Microbiol.

[3] She W, Ye W, Shi Y, Zhou L, Zhang Z, Chen F, Qian PY (2020). A novel chresdihydrochalcone from *Streptomyces chrestomyceticus* exhibiting activity against Gram-positive bacteria. J Antibiot.

[4] Lu K, Chen Q, Xu XF, Meng Y, Lin J, Chen WM (2020). Novel benzyl phenyl sulfide derivatives as antibacterial agents against methicillin-resistant *Staphylococcus aureus*. J Antibiot.

[5] Ventola CL (2015). The antibiotic resistance crisis: part 1: causes and threats. Peer-reviewed J Form Manag.

[6] Özgeriş B (2021). Design, synthesis, characterization, and biological evaluation of nicotinoyl thioureas as antimicrobial and antioxidant agents. J Antibiot.

[7] Gavade SN, Markad VL, Kodam KM, Shingare MS, Mane DV (2012). Synthesis and biological evaluation of novel 2,4,6-triazine derivatives as antimicrobial agents. Bioorg Med Chem Lett.

[8] Solankee A, Kapadia K, Ana C, Soković M, Doytchinova I, Geronikaki A (2010). Synthesis of some new S-triazine based chalcones and their derivatives as potent antimicrobial agents. Eur J Med Chem.

[9] Patel RV, Patel AB, Kumari P, Chikhalia KH (2012). Synthesis of novel 3-(5-sulfanyl-1,3,4-oxadiazol-2-yl)-2*H*-chromen-2-one condensed *s*-triazinyl piperazines and piperidines as antimicrobial agents. Med Chem Res.

[10] Singh UP, Bhat HR, Gahtori P (2012). Antifungal activity, SAR and physicochemical correlation of some thiazole-1,3,5-triazine derivatives. J Mycologie Médicale.

[11] Kumar S, Bhat HR, Kumawat MK, Singh UP (2013). Design and one-pot synthesis of hybrid thiazolidin-4-one-1, 3, 5-triazines as potent antibacterial agents against human disease causing pathogens. New J Chem.

[12] Bhat HR, Gupta SK, Singh UP (2012). Discovery of potent, novel antibacterial hybrid conjugates from 4-aminoquinoline and 1, 3, 5-triazine: design, synthesis and antibacterial evaluation. RSC Adv.

[13] Bhat HR, Pandey PK, Ghosh SK, Singh UP (2013). Development of 4-aminoquinoline-1,3,5-triazine conjugates as potent antibacterial agent through facile synthetic route. Med Chem Res.

[14] Modh RP, De Clercq E, Pannecouque C, Chikhalia KH (2014). Design, synthesis, antimicrobial activity and anti-HIV activity evaluation of novel hybrid quinazolinetriazine derivatives. J Enzyme Inhib Med Chem.

[15] Modh RP, Patel AC, Chikhalia KH (2013). Design, synthesis, antibacterial, and antifungal studies of novel 3-substituted coumarinyl-triazine derivatives. Heterocycl Commun.

[16] Kumar A, Menon SK (2009). Fullerene derivatized s-triazine analogues as antimicrobial agents. Eur J Med Chem.

[17] Saleh M, Abbott S, Perron V, Lauzon C, Penney C, Zacharie B (2010). Synthesis and antimicrobial activity of 2-fluorophenyl-4, 6-disubstituted [1,3,5] triazines. Bioorg Med Chem Lett.

[18] Srinivas K, Srinivas U, Rao VJ, Bhanuprakash K, Kishore KH, Murty U (2005). Synthesis and antibacterial activity of 2,4,6-tri substituted s-triazines. Bioorg Med Chem Lett.

[19] Shanmugam M, Narayanan K, Chidambaranathan V, Kabilan S (2013). Synthesis, spectral characterization and antimicrobial studies of novel s-triazine derivatives. Acta A Mol Biomol Spectrosc.

[20] Sun X, Cao Z, Sun Y (2009). *N*-chloro-alkoxy-s-triazine-based antimicrobial additives: Preparation, characterization, and antimicrobial and biofilm-controlling functions. Ind Eng Chem Res.

[21] Ma X, Tan ST, Khoo CL, Sim HM, Chan LW, Chui WK (2011). Synthesis and antimicrobial activity of *N*^1^-benzyl or *N*^1^-benzyloxy-1,6-dihydro-1,3,5-triazine-2,4-diamines. Bioorg Med Chem Lett.

[22] Cascioferro S, Parrino B, Spanò V, Carbone A, Montalbano A, Barraja P, Diana P, Cirrincione G (2017). An overview on the recent developments of 1,2,4-triazine derivatives as anticancer compounds. Eur J Med Chem.

[23] Kciuk M, Marciniak B, Celik I, Zerroug E, Dubey A, Sundaraj R, Mujwar S, Bukowski K, Mojzych M, Kontek R (2023). Pyrazolo[4,3-*e*]tetrazolo[1,5-*b*][1,2,4]triazine sulfonamides as an important scaffold for anticancer drug discovery-in vitro and in silico evaluation. Int J Mol Sci.

[24] Gornowicz A, Szymanowska A, Mojzych M, Bielawski K, Bielawska A (2020). The effect of novel 7-methyl-5-phenyl-pyrazolo[4,3-*e*]tetrazolo[4,5-*b*][1,2,4]triazine sulfonamide derivatives on apoptosis and autophagy in DLD-1 and HT-29 colon cancer cells. Int J Mol Sci.

[25] Bernat Z, Szymanowska A, Kciuk M, Kotwica-Mojzych K, Mojzych M (2020). Review of the synthesis and anticancer properties of pyrazolo[4,3-*e*][1,2,4]triazine derivatives. Molecules.

[26] Hermanowicz JM, Szymanowska A, Sieklucka B, Czarnomysy R, Pawlak K, Bielawska A, Bielawski K, Kalafut J, Przybyszewska A, Surazynski A (2021). Exploration of novel heterofused 1,2,4-triazine derivative in colorectal cancer. J Enzym Inhib Med Chem.

[27] Venkatesan AM, Chen Z, Dehnhardt CM, Dos Santos O, Delos Santos EG, Zask A, Verheijen JC, Kaplan JA, Richard DJ, Ayral-Kaloustian S, Mansour TS, Gopalsamy A, Curran KJ, Shi M. Triazine compounds as PI3 kinase and MTOR inhibitors. Current Patent Assignee: PFIZER - US2017/224696, 2017, A9.

[28] Lu Y, Chen J, Xiao M, Li W, Miller DD (2012). An overview of tubulin inhibitors that interact with the colchicine binding site. Pharm Res.

[29] Cheng X-C, Wang Q, Fang H, Xu W-F (2008). Role of sulfonamide group in matrix metalloproteinase inhibitors. Curr Med Chem.

[30] Bouchain G, Delorme D (2003). Novel hydroxamate and anilide derivatives as potent histone deacetylase inhibitors: Synthesis and antiproliferative evaluation. Curr Med Chem.

[31] Mojzych M, Tarasiuk P, Kotwica-Mojzych K, Rafiq M, Seo S-Y, Nicewicz M, Fornal E (2016). Synthesis of chiral pyrazolo[4,3-*e*][1,2,4]triazine sulfonamides with tyrosinase and urease inhibitory activity. J Enzym Inhib Med Chem.

[32] Sciú ML, Sebastián-Pérez V, Martinez-Gonzalez L, Benitez R, Perez DI, Pérez C, Campillo NE, Martinez A, Moyano EL (2018). Computer-aided molecular design of pyrazolotriazines targeting glycogen synthase kinase 3. J Enzym Inhib Med Chem.

[33] Mojzych M, Šubertová V, Bielawska A, Bielawski K, Bazgier V, Berka K, Gucký T, Fornal E, Kryštof V (2014). Synthesis and kinase inhibitory activity of new sulfonamide derivatives of pyrazolo[4,3-*e*][1,2,4]triazines. Eur J Med Chem.

[34] Filhol O, Cochet C (2009). Protein Kinase CK2 in Health and Disease. Exp.

[35] Kciuk M, Gielecińska A, Mujwar S, Mojzych M, Marciniak B, Drozda R, Kontek R (2022). Targeting carbonic anhydrase IX and XII isoforms with small molecule inhibitors and monoclonal antibodies. J Enzym Inhib Med Chem.

[36] Kciuk M, Mujwar S, Szymanowska A, Marciniak B, Bukowski K, Mojzych M, Kontek R (2022). Preparation of novel pyrazolo[4,3-*e*]tetrazolo[1,5-*b*][1,2,4]triazine sulfonamides and their experimental and computational biological studies. Int J Mol Sci.

[37] Wan Y, Fang G, Chen H, Deng X, Tang Z (2021). Sulfonamide derivatives as potential anti-cancer agents and their SARs elucidation. Eur J Med Chem.

[38] Elgemeie GH, Mohamed-Ezzat RA. New Strategies Targeting Cancer Metabolism, **2022**, pp. 1–619. Amsterdam: Elsevier. ISBN: 978–0–12–821783–2. DOI:10.1016/B978-0-12-821783-2.00010-8.

[39] Elgemeie GH, Azzam RA, Zaghary WA, Aly AA, Metwally NH, Sarhan MO, Abdelhafez EM, Elsayed RE. *N*-Sulfonated-*N*-heterocycles: synthesis, chemistry, and biological applications, **2022**, pp. 1–507. Amsterdam: Elsevier. ISBN: 978–0–12–822179–2. DOI:10.1016/B978-0-12-822179-2.00004-5.

[40] Mohamed-Ezzat RA, Kariuki BM, Elgemeie GH (2023). Unexpected products of the reaction of cyanoacetylhydrazones of aryl/heteryl ketones with hydrazine: a new route to aryl/heteryl hydrazones, X-ray structure, and in vitro anti-proliferative activity against NCI 60-cell Line Panel. Egypt J Chem.

[41] Mohamed-Ezzat RA, Hashem AH, Dacrory S (2023). Synthetic strategy towards novel composite based on substituted pyrido[2,1-*b*][1,3,4]oxadiazine-dialdehyde chitosan conjugate with antimicrobial and anticancer activities. BMC Chem.

[42] Elgemeie GH, Salah AM, Abbas NS, Hussein HA, Mohamed RA (2017). Pyrimidine non-nucleoside analogs: A direct synthesis of a novel class of *N*-substituted amino and *N*-sulfonamide derivatives of pyrimidines. Nucleosides Nucleotides.

[43] Elgemeie GH, Mohamed RA, Hussein HA, Jones PG (2015). Crystal structure of *N*-(2-amino-5-cyano-4-methylsulfanyl-6-oxo-1,6-dihydropyrimidin-1-yl)-4-bromobenzenesulfonamide dimethylformamide monosolvate. Acta Crystallogr E Crystallogr Commun.

[44] Alaoui S, Dufies M, Driowya M, Demange L, Bougrin K, Robert G, Auberger P, Pages G, Benhida R (2017). Synthesis and anti-cancer activities of new sulfonamides 4-substituted-triazolyl nucleosides. Bioorg Med Chem Lett.

[45] Zain-Alabdeen AI, El-Moselhy TF, Sharafeldin N, Angeli A, Supuran CT, El-Hamamsy MH (2022). Synthesis and anticancer activity of new benzensulfonamides incorporating s-triazines as cyclic linkers for inhibition of carbonic anhydrase IX. Sci Rep.

[46] Kumar S, Rulhania S, Jaswal S, Monga V (2021). Recent advances in the medicinal chemistry of carbonic anhydrase inhibitors. Eur J Med Chem.

[47] Manzoor S, Petreni A, Raza MK, Supuran CT, Hoda N (2021). Novel triazole-sulfonamide bearing pyrimidine moieties with carbonic anhydrase inhibitory action: Design, synthesis, computational and enzyme inhibition studies. Bioorg Med Chem Lett.

[48] https://go.drugbank.com/drugs/DB05245.

[49] https://go.drugbank.com/drugs/DB06147.

[50] https://go.drugbank.com/drugs/DB00576.

[51] Moskalik MY (2023). Sulfonamides with Heterocyclic Periphery as Antiviral Agents. Molecules.

[52] Elgemeie GH, Mohamed RA (2014). Application of dimethyl *N*-cyanodithioiminocarbonate in synthesis of fused heterocycles and in biological chemistry. Heterocycl Commun.

[53] Elgemeie GH, Mohamed RA (2014). Recent trends in synthesis of five- and six-membered heterocycles using dimethyl *N*-cyanodithioiminocarbonate. Heterocycl Commun.

[54] Elgemeie GH, Mohamed RA (2023). Discovery and synthesis of novel bio-isostere of purine analogues inhibiting SARS-CoV-2. Egypt J Chem.

[55] Elgemeie GH, Alkhursani SA, Mohamed RA (2019). New synthetic strategies for acyclic and cyclic pyrimidinethione nucleosides and their analogues. Nucleosides Nucleotides.

[56] Elgemeie GH, Mohamed RA (2019). Microwave chemistry: synthesis of purine and pyrimidine nucleosides using microwave radiation. J Carbohydr Chem.

[57] Elgemeie GH, Salah AM, Abbas NS, Hussein HA, Mohamed RA (2017). Nucleic acid components and their analogs: design and synthesis of novel cytosine thioglycoside analogs. Nucleosides Nucleotides.

[58] Elgemeie GH, Salah AM, Mohamed RA, Jones PG (2015). Crystal structure of (*E*)-2-amino-4-methylsulfanyl-6-oxo-1-{[(thiophen-2-yl)methylidene]amino}-1,6-dihydropyrimidine-5-carbonitrile. Acta Crystallogr E Crystallogr Commun.

[59] Mohamed-Ezzat RA, Elgemeie GH, Jones PG (2021). Crystal structures of (*E*)-2-amino-4-methyl-sulfanyl-6-oxo-1-(1-phenyl-ethyl-idene-amino)-1,6-di-hydro-pyrimidine-5-carbo-nitrile and (*E*)-2-amino-4-methyl-sulfanyl-6-oxo-1-[1-(pyridin-2-yl)ethyl-idene-amino]-1,6-di-hydro-pyrimidine-5-carbo-nitrile. Acta Crystallogr E Crystallogr Commun.

[60] Mohamed-Ezzat RA, Elgemeie GH, Jones PG (2024). An unexpected tautomer: synthesis and crystal structure of *N*-[6-amino-4-(methyl­sulfan­yl)-1,2-di­hydro-1,3,5-triazin-2-yl­idene]benzenesulfonamide. Acta Crystallogr E Crystallogr Commun.

[61] Kumara JA, Amarnatha DJ, Kumarb PS, Kaushika CS, Varghesea ME, Saravanan A (2018). Mass transfer and thermodynamic analysis on the removal of naphthalene from aqueous solution using oleic acid modified palm shell activated carbon. Desalin Water Treat.

[62] Kumar JA, Sathish S, Krithiga T, Praveenkumar TR, Lokesh S, Prabu D, Annam Renita A, Prakash P, Rajasimman M (2022). A comprehensive review on bio-hydrogen production from brewery industrial wastewater and its treatment methodologies. Fuel.

[63] Kumar JA, Krithiga T, Narendrakumar G, Prakash P, Balasankar K, Sathish S, Prabu D, Purna Pushkala D, Marraiki N, Ramu AG, Choi D (2022). Effect of Ca2+ ions on naphthalene adsorption/desorption onto calcium oxide nanoparticle: Adsorption isotherm, kinetics and regeneration studies. Environ Res.

[64] http://dtp.nci.nih.gov.

[65] Monks A, Scudiero D, Skehan P, Shoemaker R, Paull K, Vistica D, Hose C, Langley J, Cronise P, Vaigro-Wolff A (1991). Feasibility of a high-flux anticancer drug screen using a diverse panel of cultured human tumor cell lines. J Natl Cancer Inst.

[66] Boyd MR, Teicher BA (1997). Cancer Drug Discovery and Development, 2.

[67] Shoemaker RH (2006). The NCI60 human tumor cell line anticancer drug screen. Nat Rev Cancer.

[68] Boyd MR, Paull KD (1995). Some practical considerations and applications of the national cancer institute in vitro anticancer drug discovery screen. Drug Dev Res.

[69] Scótt AC, Collee JG, Duguid JP, Fraser AG, Marmion BP (1989). Laboratory control of antimicrobial therapy. Practical Medical Microbiology.

